# Exploring CT pixel and voxel size effect on anatomic modeling in mandibular reconstruction

**DOI:** 10.1186/s41205-024-00223-0

**Published:** 2024-06-26

**Authors:** Maariyah Ahmed, Myra Garzanich, Luigi E. Melaragno, Sarah Nyirjesy, Natalia Von Windheim, Matthew Marquardt, Michael Luttrull, Nathan Quails, Kyle K. VanKoevering

**Affiliations:** 1 Center for Design and Manufacturing Excellence, The Ohio State University, Columbus, OH USA; 2 Department of Biomedical Engineering, The Ohio State University College of Engineering, Columbus, OH USA; 3 Department of Otolaryngology–Head and Neck Surgery, The Ohio State University, 460 W 10th Ave 5th Floor Clinic, Columbus, OH 43220 USA; 4 Department of Radiology, The Ohio State University Wexner Medical Center, Columbus, OH USA

**Keywords:** Anatomic modeling, CT scan resolution, Head and neck surgery, Mandibular reconstruction, Pixel size, Slice thickness, Patient-specific modeling

## Abstract

**Background:**

Computer-aided modeling and design (CAM/CAD) of patient anatomy from computed tomography (CT) imaging and 3D printing technology enable the creation of tangible, patient-specific anatomic models that can be used for surgical guidance. These models have been associated with better patient outcomes; however, a lack of CT imaging guidelines risks the capture of unsuitable imaging for patient-specific modeling. This study aims to investigate how CT image pixel size (X-Y) and slice thickness (Z) impact the accuracy of mandibular models.

**Methods:**

Six cadaver heads were CT scanned at varying slice thicknesses and pixel sizes and turned into CAD models of the mandible for each scan. The cadaveric mandibles were then dissected and surface scanned, producing a CAD model of the true anatomy to be used as the gold standard for digital comparison. The root mean square (RMS) value of these comparisons, and the percentage of points that deviated from the true cadaveric anatomy by over 2.00 mm were used to evaluate accuracy. Two-way ANOVA and Tukey-Kramer post-hoc tests were used to determine significant differences in accuracy.

**Results:**

Two-way ANOVA demonstrated significant difference in RMS for slice thickness but not pixel size while post-hoc testing showed a significant difference in pixel size only between pixels of 0.32 mm and 1.32 mm. For slice thickness, post-hoc testing revealed significantly smaller RMS values for scans with slice thicknesses of 0.67 mm, 1.25 mm, and 3.00 mm compared to those with a slice thickness of 5.00 mm. No significant differences were found between 0.67 mm, 1.25 mm, and 3.00 mm slice thicknesses. Results for the percentage of points deviating from cadaveric anatomy greater than 2.00 mm agreed with those for RMS except when comparing pixel sizes of 0.75 mm and 0.818 mm against 1.32 mm in post-hoc testing, which showed a significant difference as well.

**Conclusion:**

This study suggests that slice thickness has a more significant impact on 3D model accuracy than pixel size, providing objective validation for guidelines favoring rigorous standards for slice thickness while recommending isotropic voxels. Additionally, our results indicate that CT scans up to 3.00 mm in slice thickness may provide an adequate 3D model for facial bony anatomy, such as the mandible, depending on the clinical indication.

## Introduction

3-dimensional (3D) printing has played an increasingly significant role in healthcare. An important use for this technology is the creation of patient-specific anatomic models that can be used for preoperative planning and intraoperative guidance of surgical procedures. The use of 3D printed, patient-specific models in the operating room provides surgeons with a comprehensive and tangible, 360-degree view of their patient’s anatomy and has been associated with decreased operating times while improving patient outcomes [1]. At a select number of institutions, certain specialties–such as craniomaxillofacial reconstruction surgery [2], pediatric cardiology [3], and renal system surgery [4]--have adopted 3D printed anatomic modeling as standard practice.

To produce a 3D model, a patient’s cross-sectional imaging (typically computed tomography (CT) or magnetic resonance imaging (MRI)) is uploaded to a segmentation software and trained personnel work through a standardized segmentation procedure to isolate the patient’s target anatomy. Initially, a threshold based on the Hounsfield units for bone or soft tissue is applied to the images to isolate the desired anatomy. Then, non-relevant anatomic structures and artifacts from the CT scan are removed, resulting in a rough 3D rendering of the target anatomy. The model is then digitally sculpted to smooth sharp edges and eliminate internal geometries that would cause printing failures [2]. Once all digital edits are completed, the 3D model is printed and post-processed accordingly. Stereolithography (SLA) printing is commonly used for medical 3D printing due to the ability to sterilize the material prior to use in the operating room (OR) [5].

**Fig. 1:**
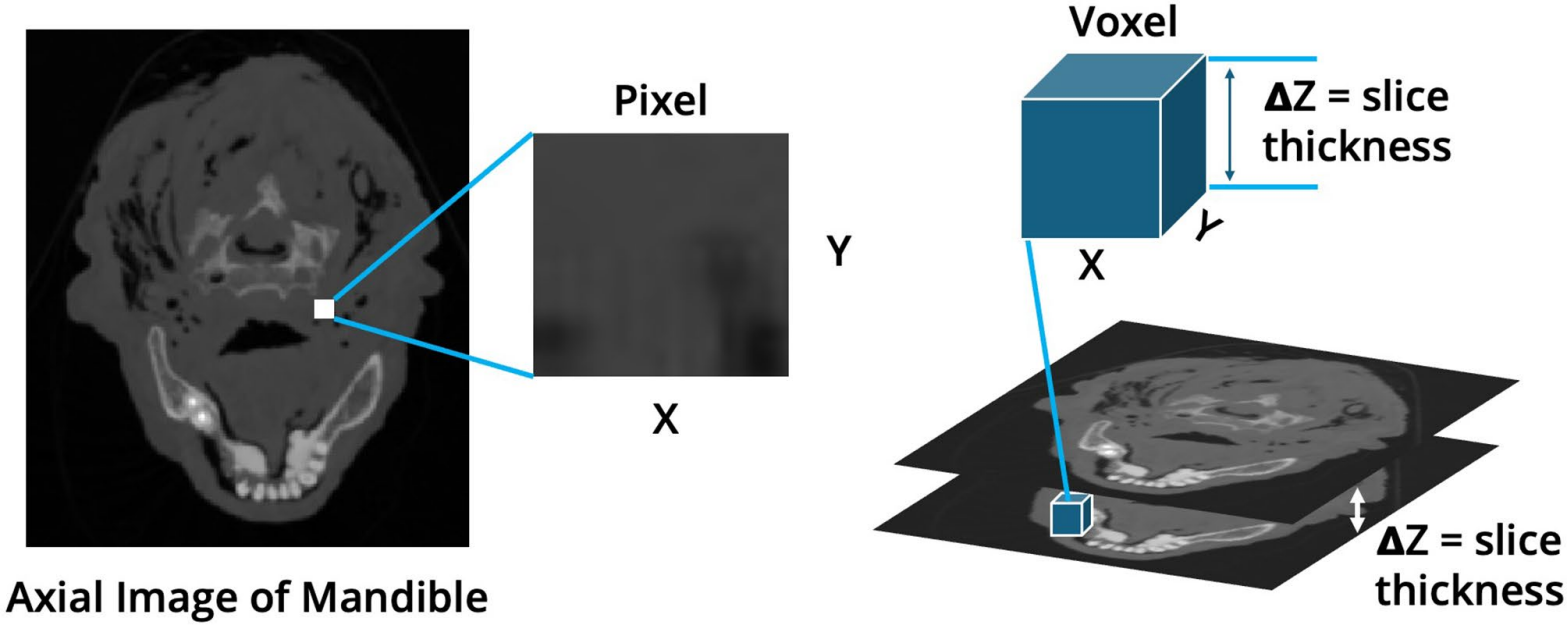
Illustration of CT scan pixels, slice thickness, and voxels

The accuracy of a 3D model relative to the patient’s true anatomy ultimately hinges on the source imaging data. Thus, the resolution of the CT scan used for modeling plays an important role in the accuracy of the final 3D printed model when compared to the patient’s true anatomy [6–10]. CT imaging data consists of a series of 2-dimensional images ‘stacked’ on top of each other in the axial plane. The distance between two-dimensional images is termed slice thickness. The resolution of the two-dimensional images, termed pixel size, is defined by the Modulation Transfer Function (MTF) in the reconstruction (X-Y) plane and the Z plane. The MTF can be obtained by taking either the Fourier Transform (FT) of the Point Spread Function (PSF) in the X-Y plane or the FT of the Slice Sensitivity Profile (SSP) in the Z-direction to obtain the limiting spatial resolutions along those dimensions, respectively [11]. In practice, the MTF is not routinely calculated to assess the resolution; however, a more common surrogate is the CT image ‘voxel’ which is defined by the respective X-Y-and Z dimensions (Fig. 1) [11].

Using CT images with proper resolution for anatomic modeling is critically important to accurately represent the patient’s anatomy. Standards have been developed to help inform clinicians, point-of-care 3D printing services, and private entities what parameters should be used when acquiring CT images for medical modeling and 3D printing purposes. Many of these standards recommend minimizing slice thickness, yet there is no consensus on the maximum allowable slice thickness [6–8]. For example, the RSNA’s Guidelines for Medical 3D Printing (2018) suggest that the smallest anatomical feature of interest should appear in at least three slices of the CT scan used for modeling. Thus, if the smallest anatomical feature of interest were 3 mm, then the largest acceptable slice thickness would be 1 mm [6]. On the other hand, Bibb and Winder’s review article regarding CT imaging for medical modeling (2010) recommends a slice thickness of 0.5 –1 mm for maxillofacial surgical applications and states that slice thickness should never exceed 2 mm in any clinical scenario [8]. Lacking objective data, the only justification for these recommendations seems to be that larger slice thickness could lead to potential data loss and ‘stair-stepping’ artifact. Importantly, these guidelines neglect to offer recommendations for pixel size resolution. The only mention of spatial resolution in the X-Y dimension comes from the suggestion that voxels should be isometric [equal X-Y dimensions], with no comment on pixel size and a lack of data demonstrating how pixel size impacts 3D resolution. More rigorous research is needed to define proper standards in this field, especially as it relates to resolution in the X-Y dimension.

Our previous research, published by Ahmed and Melaragno et al. (2023), investigated the effects of slice thickness on the resulting accuracy of 3D printed mandible models and found that requiring the highest possible CT scan resolution in the Z-dimension may be unnecessary [2]. In this previous study, slice thickness was the only scan parameter under investigation and pixel size was held constant. However, most CT scans acquired in the clinic vary significantly in the X-Y dimension as well as the Z-dimension. As such, it is important to determine a standard for both pixel size and slice thickness to better control and predict the accuracy of a patient-specific 3D model consistently.

This study aims to determine the effect of CT pixel size and slice thickness resolution on 3D model accuracy. Mandibular bony anatomy was used as the target reference since 3D modeling is frequently used for craniofacial applications and the mandible represents a discrete 3D object that can be readily dissected and measured with a gold-standard method.

## Methods

This study was conducted as a cross-sectional cadaveric cohort. The Ohio State University’s Division of Anatomy and Body Donation program provided six formalin-fixed cadaver heads (no IRB exemption required). A typical modeling process for patient-specific 3D printed anatomical models was followed with the cadaveric specimens to compare model accuracy across various CT resolutions.

### CT scan acquisition and data collection

A Philips/Ingenuity 128 slice CT Scanner (Philips Healthcare, Cleveland, OH) was used to scan the cadaveric heads. A positioning aid was used to fixate the heads, ensuring stability and consistency in the scanning process. Several different scan acquisitions were performed and replicated across each head with a field of view ranging from 164 mm to 676 mm, a KVP of 120 kV, a tube current of 200 mAs, a pitch of 0.298, and a matrix size of 512 × 512. Each scan resolution is defined in Table 1 ordered by voxel size. In total, 11 different configurations of slice thickness and pixel size were included in the analysis [Table 1]. Settings were chosen that represent typical values used for clinically indicated head and neck scans.

**Table 1:** Average RMS values and percent of points deviating by more than 2 mm for comparisons between CAD models and cadaveric anatomy at each CT scan resolution. * Indicates data used from Ahmed, Melaragno et al. [2].

VOXEL SIZE (mm^3^)	Z DIM (mm)	X/Y DIM (mm)	Average RMS [95% CI] (mm)	Average % of Points Deviating > 2 mm [95% CI]
0.0686	0.67	0.32	0.8259 [0.7739–0.8779]	1.2183 [0.1987–2.2378]
0.3072	3.00	0.32	0.9199 [0.8817–0.9581]	2.5127 [0.2005–4.4015]
0.4483*	0.67	0.818	0.8655 [0.7696–0.9615]	1.2748 [-0.3345-2.8842]
0.7031	1.25	0.75	0.8958 [0.8694–0.9222]	2.3010 [0.9377–4.0877]
0.8364*	1.25	0.818	0.8777 [0.8478–0.9076]	2.1181 [0.0529–4.1833]
1.6875	3.00	0.75	0.9767 [0.9402–1.0131]	3.8298 [2.2349–5.4247]
2.0073*	3.00	0.818	1.0104 [0.8802–1.1407]	2.8597 [0.3507–5.3688]
3.0000	3.00	1.00	1.0054 [0.9892–1.0217]	4.4367 [1.1837–7.6898]
3.3456*	5.00	0.818	1.3771 [0.9783–1.7759]	8.2031 [0.6050–15.8012]
5.2272	3.00	1.32	1.0186 [0.9775–1.0598]	4.8682 [2.4435–7.2929]
8.7120	5.00	1.32	1.3774 [1.0430–1.7118]	10.4424 [5.5659–15.3188]

### Segmentation and CAD processing of CT scans

The acquired CT scans captured the entire cadaveric head and neck anatomy. Once the CT scans were completed, Materialise Mimics Innovation Suite (Materialise, Leuven, Belgium) software was used to isolate the mandible. Hounsfield unit thresholding was used to isolate the bony mandibular anatomy, and manual steps were taken to remove any CT artifacts and ensure the standardized bone threshold did not remove any target anatomy. A resulting CAD model was produced for each set of scans, producing eleven models per cadaveric head for 66 unsculpted models total. These models were then smoothed and fixed in Materialise 3-Matic (Materialise, Leuven, Belgium) software. This software was used to remove interior structures, fill model holes, and perform local smoothing, known as ‘digital sculpting’ [2]. Although digital sculpting processes aid in the printability of the model, they can also potentially lead to sources of inaccuracies due to their subjective nature [12]. However, previous studies have shown that overall, digital sculpting significantly improves the model’s accuracy [2]. Each personnel of the modeling team underwent in-house training on Materialise Mimics and 3-Matic before study methodology was performed to ensure reliability and proper technique in model production processes.

### Cadaveric dissection and surface scanning

After completing the necessary CT scans of the cadaveric heads, dissection and isolation of the mandible as well as surface scanning of dissected mandibles was performed [2]. The isolated mandibles were surface scanned with a high resolution and accuracy (0.1 mm and 0.05 mm) Artec Space Spider scanner (Artec3D, Luxembourg) to produce digital 3D models. This digital rendering of the cadaver bones served as the gold standard for accuracy compared to the CT-derived models.

### Model accuracy data collection

**Fig. 2:**
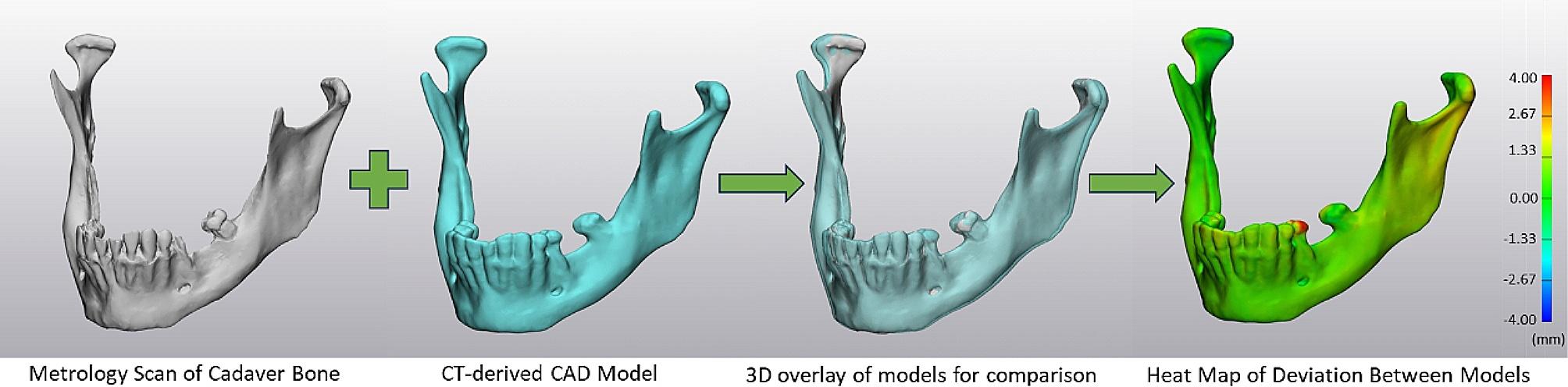
Digital alignment of cadaver bone surface scans and CT-derived CAD models which utilizes an n-point registration followed by a global alignment algorithm

The CAD model produced from the surface scan was then digitally aligned with the CAD models produced from the CT scans of varying slice thickness and pixel sizes for a given mandible. Upon alignment, a part comparison analysis quantified the difference between the surface geometry of the models (Fig. 2). This point-based analysis calculated the root mean squared (RMS), the mean, and the standard deviation for distance between the two models for each comparison. The RMS value quantified the accuracy of each CT-derived model in comparison to the native cadaveric anatomy [2, 13]. Additionally, the percentage of points deviating from the cadaveric gold standard beyond 2 mm, a standard clinically accepted error in orthognathic surgical planning [14], was recorded.

### Independent & dependent variables

Both slice thickness and voxel size were explored as independent variables, considering changes in the X-Y, and Z dimensions. In all cases, RMS and percentage of points deviating beyond 2 mm represented the dependent variable and described the model’s accuracy compared to the surface-scanned cadaveric bone. A larger RMS and percent deviation represent greater discrepancy between the two compared models.

### Data analyses

Microsoft Excel was used to calculate the average RMS and average percentage of points that deviated beyond 2 mm for each scan level. Statistical analyses were performed using SPSS Statistics (IBM, Chicago, IL). A two-way analysis of variance (ANOVA) comparing RMS values was completed with all scan data to determine significant differences based on slice thickness or pixel size. A two-way ANOVA was also performed using the average percent deviation beyond 2 mm at all scan levels. Analysis was supplemented with Tukey-Kramer post-hoc testing to make comparisons based on slice thickness and pixel size alone (α = 0.05).

## Results

A total of 66 comparisons were made between resulting CAD models from various scan resolutions and the native cadaveric anatomy. The average RMS values and the percentage of points that deviated from the cadaveric anatomy by greater than 2 mm were recorded for each of the CT scan resolutions (Table 1).

### Impact of slice thickness and pixel size

**Fig. 3:**
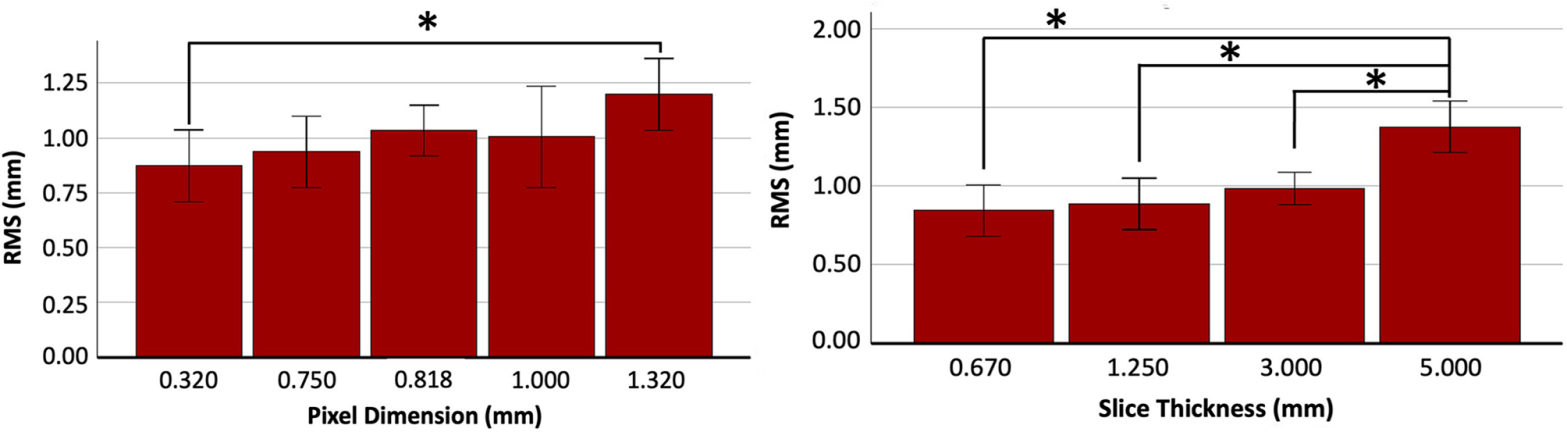
Tukey-Kramer post-hoc testing after two-way ANOVA isolating the effect of pixel size on the RMS values (Left) and isolating the effect of slice thickness on RMS values (right). Clinically, a typical CT neck will have pixel sizes of approximately 0.80 mm and slice thickness of 3.00 mm. * Indicates significance between groups at *p* < 0.05. Error bars represent 95% confidence intervals

Two-way ANOVA of all data demonstrated significant difference in average RMS based on slice thickness (*P* = 0.001), but not based on pixel size. There was also no significant interaction between slice thickness and pixel size. Tukey-Kramer post hoc testing was performed for pixel size and slice thickness. For pixel size, there was significance observed only between scans with a pixel of 0.32 mm and 1.32 mm (*P* = 0.049) (Fig. 3). Tukey-Kramer post-hoc analysis between slice thicknesses revealed significantly smaller RMS values for scans with a slice thickness of 0.67 mm (*P* < 0.001), 1.25 mm (*P* < 0.001), and 3.00 mm (*P* < 0.001) when compared to those with a slice thickness of 5.00 mm (Fig. 3). No significant differences were found between 0.67 mm, 1.25 mm, and 3.00 mm scans.

**Fig. 4:**
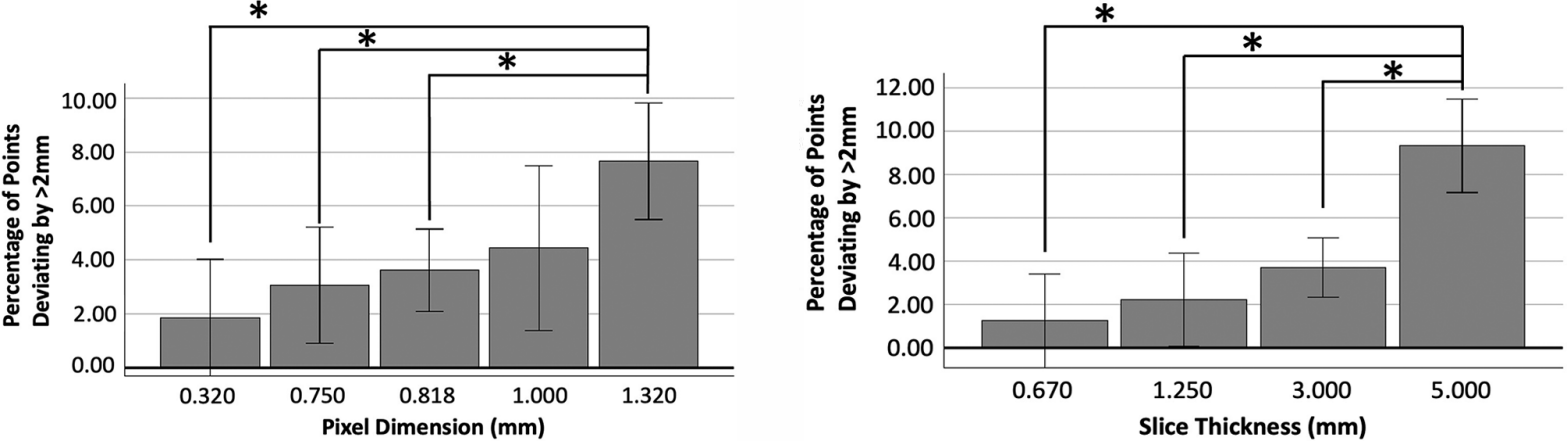
Tukey-Kramer post-hoc testing after two-way ANOVA isolating the effect of pixel size on the percentage of points deviating from the true anatomy by more than 2.00 mm (Left) and isolating the effect of slice thickness on percentage of points deviating from the true anatomy by more than 2.00 mm (right). Clinically, a typical CT neck will have pixel sizes of approximately 0.80 mm and slice thickness of 3.00 mm.* Indicates significance between groups at *p* < 0.05. Error bars represent 95% confidence intervals

The results for the two-way ANOVA of the percentage of points deviating from cadaveric anatomy beyond 2 mm agreed with the results for average RMS, revealing significant differences based on slice thickness (*P* < 0.001) but not pixel size. There was no significant interaction between pixel size and slice thickness. When Tukey-Kramer post hoc testing was performed for pixel size and slice thickness, a significant difference was observed between a pixel dimension of 0.32 mm (*P* = 0.003), 0.75 mm (*P* = 0.031), and 0.818 mm (*P* = 0.027) when compared to 1.32 mm (Fig. 4). No differences were found between the 0.32 mm, 0.75 mm and 0.818 mm groups. It is likely that our small sample size played a role in the discrepancy between the two-way ANOVA and Tukey-Kramer analyses, and thus, would be reconciled through future studies with a larger sample size. Tukey-Kramer post-hoc analysis between slice thicknesses revealed a significantly smaller percentage of points deviating by more than 2 mm for scans with a slice thickness of 0.67 mm (*P* < 0.001), 1.25 mm (*P* < 0.001), and 3.00 mm (*P* < 0.001) when compared to those with a slice thickness of 5.00 mm (Fig. 4). Again, no differences were found between 0.67 mm, 1.25 mm, and 3.00 mm scans.

## Discussion

As highlighted above, there is no clear consensus on the scan resolution features required for 3D modeling. Many authors have advocated for different CT or MRI resolution guidelines to create an anatomically accurate 3D model. To date, there is little objective data to provide guidance on which scans are acceptable and what the expected ‘accuracy’ of the resulting 3D model may be [6–8]. This study looked to evaluate the effect of CT resolution in X-Y and Z dimensions on resulting 3D mandibular anatomy. Collectively, the data suggested that slice thickness across the clinically relevant spectrum was significantly more impactful on the accuracy of the 3D model than the X-Y dimensions of pixel size.

**Fig. 5:**
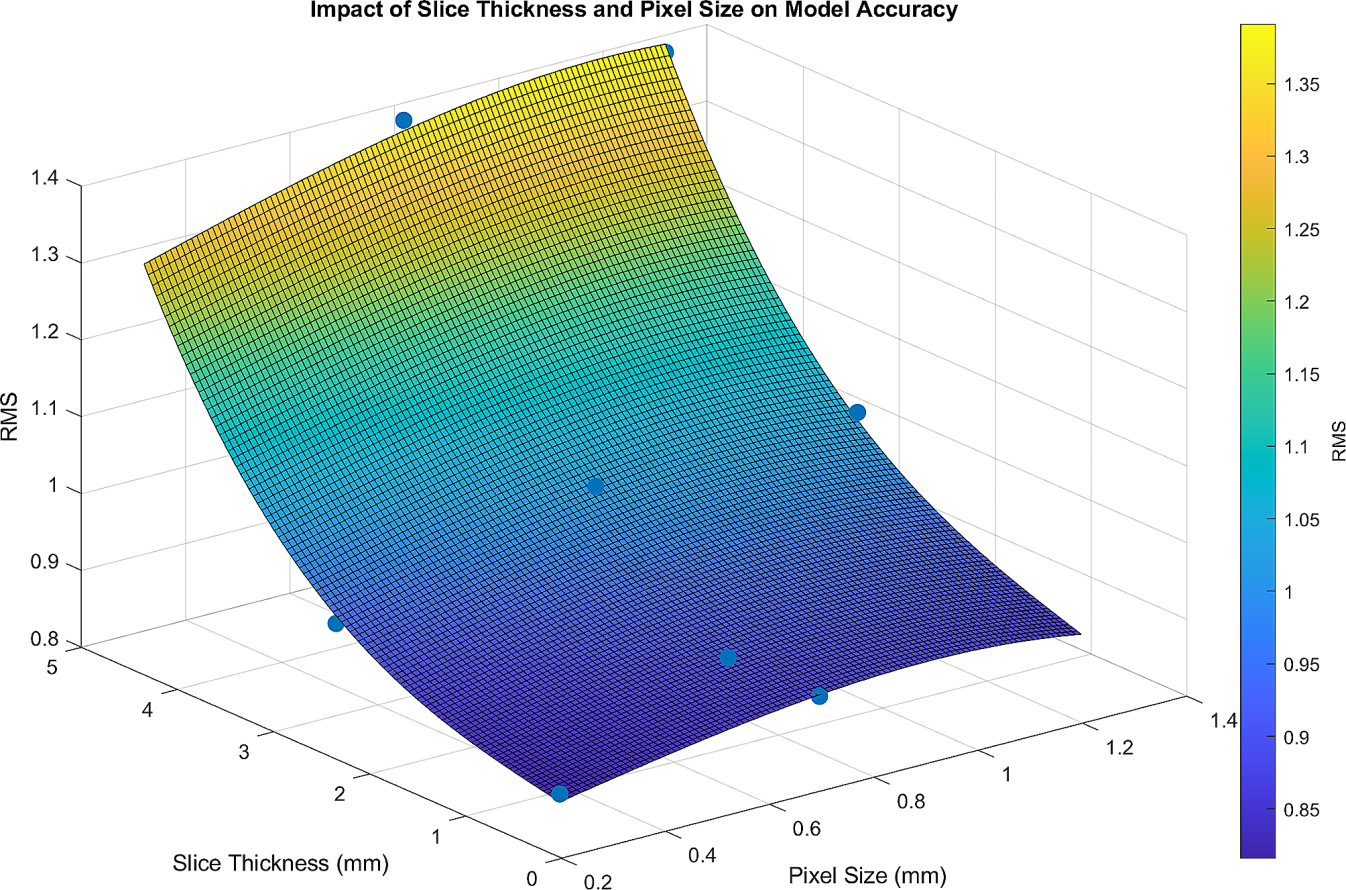
Three-dimensional plot displaying data points plotted with slice thickness and pixel size on the X and Y axes, and corresponding RMS value on the Z axis. The MATLAB surf function was used to generate a mesh overlayed on these data points to interpolate the RMS value between data points

Two-way ANOVA tests for RMS and percentage of points that deviated by more than 2.00 mm, were both significant for slice thickness, but not pixel size. While the post hoc data suggested that pixel size had some effect, this was not confirmed on the two-way ANOVA analysis. As mentioned previously, this discrepancy may be due to a small sample size. This discrepancy could also exist due to the small effect of pixel size, causing it to be overshadowed by the effect of slice thickness when both factors are compared in the same statistical test. Such a result suggests that the variance caused by pixel size is not as significant or clinically relevant as the variance caused by slice thickness. The outcome of the post-hoc tests isolating the effect of slice thickness were significant and consistent between the two measures of accuracy in this study as well as with the findings of our previous study [2]. Here, we found that anatomic models of the mandible created from CT scans with a slice thickness greater than 3.00 mm were significantly less accurate than those created from scans with a slice thickness of 3.00 mm or less. In Ahmed, Melaragno et al. (2023), one-way ANOVA testing between scans of varying slice thicknesses and constant pixel sizes revealed that models created from a slice thickness of 5.00 mm were significantly less accurate than models created from slice thicknesses of 3.00 mm or smaller. In aggregate, our results confirm that slice thickness has a larger influence on subsequent 3D model accuracy and reliability than the pixel size (Fig. 5) and reinforce the findings established by Ahmed, Melaragno et al. (2023) that 3.00 mm in slice thickness is an important threshold for ensuring model accuracy [2].

One reason that slice thickness may have a larger impact on the accuracy of a model than pixel size is because slice thickness tends to vary on a larger scale than pixel size. In this study, the range for pixel size was only 1.00 mm while the range for slice thicknesses was 4.33 mm. While clinically representative, larger variation in values of slice thickness leaves more opportunity for slice thickness to have an impact on model accuracy. Additionally, in the Z (detector) direction, you can reconstruct the image using the data from a single row of detectors (the smallest slice reconstruction). This enables the visualization of smaller objects. Because the Z-dimension is in the detector direction, identification of small features relies more heavily on the feature filling the vertical (Z) space of the voxel. With higher slice thickness, these features get lost due to signal averaging over a larger distance. In the X-Y plane, resolution is limited by the detector element size, and the data can be ‘blurred’ by the kernel filters applied. Thus, since the Z-direction signal does not have blurring from the reconstruction and did not reach the detector element size limit in terms of the slice thickness, the impact of slice thickness is more pronounced [11]. Looking at different reconstruction filters and their effect on 3D model accuracy as it relates to the X-Y plane is an area for future research.

It is also important to note that clinically, pixel size is typically controlled more by the field of view of the intended scan, while slice thickness can be driven by the intended target of the scan. To lower the slice thickness (and increase resolution), there is a tradeoff between increasing radiation dose or accepting greater image noise. Ideally, depending on the target anatomy for 3D modeling, the radiologist should aim for the optimal configuration of slice thickness and pixel size while limiting the radiation dose to the patient [15]. Figure 5 stands as a valuable visual tool that may be employed to determine the optimal combination of pixel size and slice thickness that achieves the requisite RMS for clinical accuracy and highlights that slice thickness really controls 3D model accuracy compared with pixel size. The figure clearly shows that slice thickness drives inaccuracies more than pixel size.

Our data provides objective validation aligning with prior guidelines that suggest creating standards for slice thickness as the primary target while recommending isotropic voxels. Additionally, our results suggest that when modeling facial bony anatomy such as the mandible, CT scans with up to 3.00 mm in slice thickness may provide an adequate 3D model depending on the clinical indication. Since 3.00 mm is the slice thickness used in routine clinical practice for neck and maxillofacial scans, our findings have the potential to positively impact patient care [16]. Achieving adequate models with 3.00 mm scans could reduce the need for repeat scans at smaller slice thickness, which comes with additional cost and radiation exposure. The radiation exposure and financial impact of using 3.00 mm scans over smaller thickness scans as well as the appropriateness of 3.00 mm slice thickness for other anatomical locations should be investigated in future research.

## Conclusion

Establishing reliable protocols for CT acquisition in the context of medical 3D modeling and printing is important to ensure model accuracy while balancing patient time, clinical cost, and radiation exposure. While our previous research has investigated the effects of CT scan slice thickness alone on the accuracy of 3D printed anatomic models, here we studied the effects of pixel size and the interaction between these two variables on accuracy. We showed that, while both slice thickness and pixel size impact a 3D model’s accuracy, slice thickness plays a significantly larger role. The data confirmed our previous finding that up to 3.00 mm slice thickness may serve as a threshold for the creation of accurate mandibular models depending on the clinical need.

## Data Availability

No datasets were generated or analysed during the current study.
